# Subacute Sclerosing Panencephalitis in a Child with Recurrent Febrile Seizures

**DOI:** 10.1155/2015/783936

**Published:** 2015-02-24

**Authors:** Ayşe Kartal, Ayşegül Neşe Çıtak Kurt, Tuğba Hirfanoğlu, Kürşad Aydın, Ayşe Serdaroğlu

**Affiliations:** ^1^Department of Child Neurology, Inonu University Faculty of Medicine, Malatya, Turkey; ^2^Department of Child Neurology, Gazi University Faculty of Medicine, 06500 Ankara, Turkey

## Abstract

Subacute sclerosing panencephalitis (SSPE) is a devastating disease of the central nervous system (CNS) caused by persistent mutant measles virus infection. The diagnosis of SSPE is based on characteristic clinical and EEG findings and demonstration of elevated antibody titres against measles in cerebrospinal fluid. Subacute sclerosing panencephalitis can have atypical clinical features at the onset. Herein, we report an unusual case of subacute sclerosing panencephalitis in a child with recurrent febrile seizures. The disease progressed with an appearance of myoclonic jerks, periodic high amplitude generalized complexes on EEG, and elevated titers of measles antibodies in cerebrospinal fluid leading to the final diagnosis of subacute sclerosing panencephalitis.

## 1. Introduction

Subacute sclerosing panencephalitis is a progressive, fatal neurodegenerative disease caused by an aberrant measles virus in the central nervous system [1]. The typical clinical presentation of subacute sclerosing panencephalitis includes behavioral and intellectual impairment followed by myoclonia and complete neurological deterioration depending on the degree of neuroanatomical structure involvement [2]. However, the initial characteristics and clinical course of the disease can be highly variable. Different types of seizures as prominent and a first symptom of subacute sclerosing panencephalitis are also a typical clinical presentation [3, 4]. In this report, we describe a child with subacute sclerosing panencephalitis who had history of recurrent febrile seizures.

## 2. Case Report

A 5-year-old boy was admitted to the Pediatric Neurology Department with a history of recurrent febrile convulsions which were first seen at two months of age. For the last two months they were accompanied by axial myoclonic jerks, head drops, and decreased attention span. He was born at term by spontaneous normal delivery. The antenatal period was uneventful.

**Figure 1:**
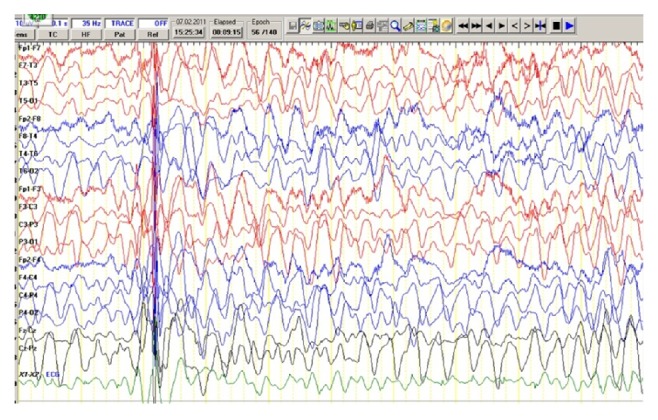
Scalp electroencephalogram showing disorganized slow background with generalized spike-wave discharges.

Milestones were achieved normally but he had not received any of his vaccinations, except for a dose of diphtheria, tetanus, pertussis vaccine. He also had past history of viral illness with skin rash and conjunctivitis, suggesting measles when he was 8 months old. His family history was negative for psychiatric or neurologic illnesses. His first febrile convulsion was observed at the age of 2 months a day after receiving a diphtheria, pertussis, and tetanus vaccination. This progressed to status epilepticus which lasted for 30 minutes. His second febrile generalized clonic seizure occurred 2 months later and prophylactic phenobarbital therapy was initiated. Afterwards he experienced two or more generalized clonic seizures per year, occurring mainly with fever. When he was 3 years old, treatment was supplemented with valproate due to a febrile generalized status epilepticus lasting one hour. During his fourth year, multiple febrile generalized tonic-clonic seizures of variable lateralization did not benefit from phenobarbital and valproate therapy. At the age of five, brief head nodding, myoclonic jerks, and cognitive stagnation appeared. Physical examination of the patient on admission was normal for vital signs. On neurologic examination, he was conscious but had gait ataxia, intention tremor, and myoclonic jerks. Myoclonic jerks involved mostly the head, the shoulders, and the arm. Cranial nerve and fundoscopic examinations were normal. The examination of motor system, tone, power, and reflexes was unremarkable. General examination did not reveal any abnormality. Laboratory investigations showed normal values of blood counts, chemistry, and electrolytes. Urine organic acids, tandem mass, plasma lactate, pyruvate, thyroid function tests, cerebrospinal fluid analysis, and magnetic resonance imaging were all normal. Electroencephalography (EEG) revealed a slow background with periodic generalized complexes consisting of bilaterally symmetrical, high voltage slow wave complexes which did not disappear with diazepam induction (Figure 1). As EEG picture was suggestive of subacute sclerosing panencephalitis, a sample of CSF was obtained for anti-measles antibody. The cerebrospinal fluid measles immunoglobulin G titer was higher than 1 : 1000. A diagnosis of subacute sclerosing panencephalitis was made in view of myoclonus, deterioration in cognitive function, elevated cerebrospinal fluid measles antibody levels, and periodic discharges in the EEG.

## 3. Discussion

SSPE is one of the most frequent causes of progressive cognitive decline in developing countries. The disease may present with varying symptoms. Uncommonly, subacute sclerosing panencephalitis may manifest with different types of seizures, such as generalized tonic-clonic seizure and myoclonic atonic seizures [5, 6]. Here, we reported a patient who presented with a history of recurrent febrile seizures, followed by deterioration in cognitive function.

This child presented with recurrent febrile seizures and a recent onset of frequent myoclonic jerks and stagnation in his cognitive status. Although this child had a history of rash illness which was suggestive of measles and he had not received MMR vaccine, we considered that was no relationship between the seizures and SSPE.

Initially, the diagnostic possibility included Dravet syndrome, but he demonstrated neither different seizures than febrile seizures nor the characteristic cognitive deterioration, and hence those differential diagnoses were excluded. The patient developed typical features of subacute sclerosing panencephalitis-like myoclonus at 5 years of age.

In light of these findings, subacute sclerosing panencephalitis was suspected and the diagnosis of subacute sclerosing panencephalitis was confirmed by the detection of cerebrospinal fluid measles antibodies. To the best of our knowledge, even though several cases involving the association of different epileptic syndrome and subacute sclerosing panencephalitis have been reported, the association of subacute sclerosing panencephalitis and recurrent febrile seizures has not been previously reported in the literature.

The role of EEG in diagnosing both atypical and typical cases of subacute sclerosing panencephalitis has been described [7]. The classic EEG picture is characterised by periodic complexes consisting of bilaterally symmetrical, synchronous, high-voltage bursts of polyphasic, stereotyped delta waves. In the different epileptic syndromes, such as Dravet syndrome, generalized periodic epileptiform discharges can be seen [8]. Moseley et al. reported a case with Dravet syndrome and SCN1A mutation that had periodic EEG discharges [9]. In another report by Takayanagi et al., a case of a 10-month-old boy whose electroencephalogram revealed generalized periodic epileptiform discharges was subsequently found to have a de novo mutation of the SCN1A gene consistent with Dravet syndrome [10].

In conclusion, subacute sclerosing panencephalitis is a rare complication of measles infection. We strongly recommend screening for SSPE in children with new onset cognitive deterioration and myoclonic jerks. Increasing awareness of SSPE in the pediatricians and neurologists can help in the early diagnosis of the patients and prevent unnecessary investigations.
